# Editorial: Insights in clinical research in nephrology

**DOI:** 10.3389/fneph.2024.1441190

**Published:** 2024-07-24

**Authors:** Michele Provenzano, Greta Borelli, Markus Pirklbauer, Gert Mayer

**Affiliations:** ^1^ Nephrology, Dialysis and Renal Tranplant Unit, Department of Pharmacy, Health and Nutritional Sciences, University of Calabria, Rende, Italy; ^2^ Nephrology, Dialysis and Renal Tranplant Unit, Scientific Institute for Research, Hospitalization and Healthcare (IRCSS)-Azienda Ospedaliero-Universitaria di Bologna, Bologna, Italy; ^3^ Department of Internal Medicine IV - Nephrology and Hypertension, Medical University Innsbruck, Innsbruck, Austria

**Keywords:** chronic kidney disease, prevention, missing heritability, advancing age, sodium intake, SIRI index, insulin

Chronic Kidney Disease (CKD) is a clinical condition defined as abnormalities in kidney structure or function (estimated glomerular filtration rate, eGFR < 60 mL/min/1.73 m^2^, and/or albuminuria > 30 mg/g) present for at least 3 months (1). Since CKD is a full-fledged chronic disease characterized by an increased cardiovascular (CV) and renal risk (2), its prevention and prompt treatment are essential to mitigate factors that can lead to worse patient outcomes. There are three levels of prevention of CKD and each of them should be promptly applied by physicians. Primary prevention consists of preventing CKD in healthy subjects by identifying risk factors such as hypertension, diabetes (3), and obesity (4). Physicians should educate the general population on practicing physical activity and having a healthier lifestyle, such as by reducing sodium intake, normalizing potassium intake, avoiding potassium deprivation, and eating more complex carbohydrates and less saturated fat (3).

Furthermore, more attention should be paid to the detection of environmental and occupational (5) risk factors as well as congenital or acquired structural anomalies of the kidney and urinary tracts and genetic factors such as APOL1 (3). Genetic kidney diseases must be detected as soon as possible to treat patient with the proper drugs. Hill et al. summarized the multi-omics analyses that could improve our knowledge of CKD and explain the so-called “missing heritability”. The authors described precisely how epigenetic, transcriptomic, metabolomic, and proteomic research is a promising and intriguing tool for future clinical application.

Among nonmodifiable CKD risk factors, there is advancing age (3). It is important to differentiate the reduction of GFR due to CKD from the natural senescence progress of renal function in order to avoid overload of the healthcare system (Frias et al.). The authors, in this article, highlighted that elderly patients defined as non-progressors (stable eGFR over time) without proteinuria may just need to be treated by their general practitioner, whereas octogenarians with fast CKD progression (annual GFR loss more than 3-5 ml/min/1.73 m^2^) could benefit from follow up at tertiary nephrology centers. They explain the physiological phenomena on which age-related GFR decline is based: among structural changes, renal mass suffers a decrease of 1 mL/min eGFR per year in people over the age of 40 through the loss of nephrons and increased cortical thinning; among functional changes, the most important is an impaired renovascular tone with a tendency toward increased renal vasoconstriction (6). The authors also emphasized that, among elderly patients, the focus should be on controlling cardiovascular risk factors rather than strict eGFR control (7).

Secondary prevention has two main goals: slowing CKD progression and, therefore, delaying transition to dialysis. Individual therapy should be offered to patients to manage proteinuria and blood pressure.

Among dietary recommendations, one of the main therapeutic strategies is to limit sodium intake. In fact, as Kang et al. reported in the KNOW-CKD study, a positive relationship between a high-salt diet and major CV events was found in non-dialysis CKD patients. The dietary sodium intake of 1.937 Korean patients was estimated on the basis of 24h urinary sodium excretion measurement. The authors revealed that a sodium intake of >8g per day was associated with higher CV risk, which was even more profound in female patients, those with abdominal obesity, and those with lower GFR. They also underlined that proteinuria categories did not modify the association between sodium intake and CV outcome even though proteinuria is a known CKD and CV mortality-related factor (8).

Still, in the context of CV risk in CKD, there are three conditions that interact with each other and contribute to raising CV risk: malnutrition-inflammation-atherosclerosis (MIA) syndrome, CKD-related mineral bone disorder (CKD-MBD), and cardio-renal-anemia (CRA) syndrome. Inflammatory cytokines, such as tumor necrosis factor-alpha (TNF alpha), interleukin-1 (IL-1), and interleukin-6 (IL-6), play a significant role in the pathogenesis of these conditions. Wei et al. showed that the systemic inflammatory response index (SIRI) may be used as a novel inflammation marker to assess the risk of mortality in CKD patients. SIRI was calculated using a simple and inexpensive formula (neutrophil count x monocyte count)/lymphocyte count.

The survival analysis conducted on more than 9.000 CKD patients revealed a greater incidence of all-cause death and CV death among groups with higher SIRI regardless of the main traditional confounding factors. The implementation of this index in clinical practice could help nephrologists and patients in prognostic assessment.

Inflammatory cytokines play a crucial role, particularly in the context of diabetic kidney disease (DKD). According to a study conducted by Daza-Arnedo et al., mediators of inflammation such as TNF alpha and IL-6 contribute to insulin resistance by impairing translational signals generated through the binding of insulin and its receptors. Conversely, hyperglycemia leads to overexpression of type 4 toll-like receptors (TLRs) at the renal tubular level and, consequently, to interstitial infiltration by macrophages. This fact is known to correlate with the progression of CKD. The authors also showed a detailed analysis of the physiological pathway of insulin at body level and its relevant renal function, suggesting this hormone as a novel therapeutic target and an intervention measure for the control of DKD.

Tertiary prevention consists of the management of uremic symptoms and comorbidities in CKD patients such as anemia, acidosis, mineral-bone disorders, protein-energy wasting, and fluid overload.

In conclusion, CKD is a heterogeneous clinical condition that needs a multidisciplinary approach. The introduction of omics and the implementation of novel biomarkers of disease and drugs may improve the understanding of underlying pathogenetic mechanisms and how to treat patients in the proper fashion. To these aims, the role of primary, secondary, and tertiary prevention is pivotal.

## Author contributions

MiP: Writing – review & editing. GB: Writing – review & editing. MaP: Writing – review & editing. GM: Writing – review & editing.
